# From the many to the few: changes in urological cancer surgery provision

**DOI:** 10.1038/sj.bjc.6602437

**Published:** 2005-03-08

**Authors:** M Nuttall, J van der Meulen, M Emberton

**Affiliations:** 1 Clinical Effectiveness Unit, The Royal College of Surgeons of England, London WC2A 3PE, UK

A growing number of studies have shown that outcomes are likely to be improved for certain complex surgical procedures if these are performed either within high-volume hospitals or by high-volume surgeons (Begg *et al*, 1998; Birkmeyer *et al*, 2002, 2003). As a consequence, the UK National Institute of Clinical Excellence (NICE), a government-funded body that provides healthcare professionals and the public with guidance on ‘best practice’, has recommended that complex urological cancer surgery be centralised into centres serving a population of at least one million (National Institute of Clinical Excellence, 2002). Based on this population, this should result in at least 50 radical operations for prostate and bladder cancer being performed per centre per year. The number of clinicians involved in treating these patients in the centres will determine whether this also provides relatively high volume at the surgeon level. Volume-based policies such as that advocated by NICE are not without controversy however, as high volume can only be a proxy for other factors that improve healthcare outcomes (Birkmeyer, 2000; Berger *et al*, 2003).

There exist three explanations of how volume and outcome may be linked (Birkmeyer, 2000). The first and most intuitive is the ‘practice makes perfect’ explanation. This postulates that improvements in outcome result from the enhanced performance gained through increased practice and experience. This effect may act in a number of areas. For example, at the individual level, a surgeon may be able to reduce operative time and possibly blood loss. At the structural level, a hospital may be better able to implement evidence-based guidelines and enhance multidisciplinary team working. The second of these explanations has been described as the ‘selective referral’ explanation. Hospitals or surgeons who have good outcomes may attract additional referrals and thus increase their volume (Ihse, 2003). This is almost certainly the case in certain centres in the USA, for example in those that attract an international clientele. The third explanation proposes that observed effects between volume and outcome might be explained by ‘confounding’. In other words, a difference in case-mix (incorporating both comorbidity and disease severity) may exist such that high-volume hospitals or surgeons care for lower risk patients.

Most evidence on the volume–outcome relationship originates from studies performed in the US. The validity of this evidence would be stronger if more data from other countries and healthcare systems were available. The healthcare system in the UK differs from that in the US in that most British patients currently do not have much control over where they receive their surgical treatment or by whom the surgical procedure is performed, although current initiatives would be expected to change this. If a volume–outcome relationship was to be found as consistently in the UK as in the US, this would mitigate against the selective referral explanation. Confounding is less likely to play a significant role, as most studies that attempt to adjust for confounding still demonstrate a positive effect between volume and outcome (Birkmeyer, 2000). Furthermore, the implementation of volume-based policies aimed at improving surgical outcomes implies that policy makers have embraced the dominance of the practice makes perfect explanation.

Based on available evidence, it is likely that implementing a volume-based policy for complex urological cancer surgery will, on average, result in improved outcomes (Nuttall *et al*, 2004). In addition, there may be other desirable by-products such as more intensive training opportunities and easier recruitment of patients into trials. The large number of cases at high-volume centres should also ensure greater statistical precision surrounding reported patient outcomes.

However, by their nature, volume-based health policies need to be applied to an entire healthcare system in order to have the maximum desired effect. This has a number of implications. First, low-volume providers with good outcomes will be lost to the healthcare system if they are either unwilling or unable to practise within high-volume centres. Second, there may also be an incentive to increase volumes through operating on patients who may not previously have been considered for surgery. Third, newly created high-volume providers may not necessarily produce better outcomes than the low-volume providers that preceded them (Shahian and Normand, 2003). Fourth, the addition of another layer to secondary care may put pressure on effective communication and adversely affect the continuum of care (Haggerty *et al*, 2003). Finally, some patients may have a preference for locally based care regardless of the local outcomes.

While acknowledging these methodological problems, if it is taken as given that outcomes improve with increasing volumes, uncertainties remain in how to translate this effect into policy. For instance, should low-volume providers be excluded from the healthcare system or should referral only take place to those providers with high volume? Moreover, at what level should volume thresholds be set? These thresholds define the minimum number of cases a provider should perform in order to be classified as ‘high’ or ‘low’ volume. Although studies have tried to identify these thresholds, considerable variation in methodology and threshold levels has been encountered (Christian *et al*, 2003; Shahian and Normand, 2003). What is more, should these thresholds be set at the surgeon level, at the hospital level or both? Should account be taken of experience in related fields? These questions illustrate the need for continuing research to define the shape of the volume and outcome curve for differing categories of providers.

Following implementation of volume-based policies, and within the new configuration, the next challenge is to identify characteristics of both centres and surgeons associated with good outcomes so that these practices might be used in both selection and training and also emulated by others to improve outcomes even further (Begg and Scardino, 2003; Shahian and Normand, 2003). This will continue to improve the quality of care while the answer to the question of ‘how many is enough?’ remains so elusive.
